# O_2_-dependent incapacitation of the *Salmonella* pathogenicity island 1 repressor HilE

**DOI:** 10.3389/fcimb.2025.1434254

**Published:** 2025-02-18

**Authors:** Steffi Walter, Valentin Schatz, Jana Petzold, Christiane Schmidt, Stefanie Hoffmann, Jonathan Jantsch, Roman G. Gerlach

**Affiliations:** ^1^ Project Group 5, Robert Koch Institute, Wernigerode, Germany; ^2^ Institute of Clinical Microbiology and Hygiene, University Hospital Regensburg and University of Regensburg, Regensburg, Germany; ^3^ Institute for Medical Microbiology, Immunology, and Hygiene, University Hospital Cologne and Faculty of Medicine, University of Cologne, Cologne, Germany; ^4^ Center for Molecular Medicine Cologne (CMMC), University of Cologne, Faculty of Medicine and University Hospital Cologne, Cologne, Germany; ^5^ Mikrobiologisches Institut – Klinische Mikrobiologie, Immunologie und Hygiene, Universitätsklinikum Erlangen and Friedrich-Alexander-Universität (FAU) Erlangen-Nürnberg, Erlangen, Germany

**Keywords:** *Salmonella*, type three secretion, hypoxia, *Salmonella* pathogenicity island, *in vitro* model

## Abstract

For successful colonization, pathogenic bacteria need to adapt their metabolism and virulence functions to challenging environments within their mammalian hosts that are frequently characterized by low oxygen (O_2_) tensions. Upon oral ingestion, the human pathogen *Salmonella enterica* serovar Typhimurium (*S*. Typhimurium) is exposed to changing O_2_ and pH levels. Low concentrations of O_2_, which can enhance the virulence of enteroinvasive pathogens, facilitate the expression of the type three secretion system (T3SS-1) encoded by the *Salmonella* pathogenicity island 1 (SPI-1) that is critical for enteroinvasion and pathogenicity of *S.* Typhimurium. To study the impact of key environmental cues of the intestine when *Salmonella* encounter enterocytes, we established an *in vitro* growth model, which allows shifting the concentration of O_2_ from 0.5% to 11% and the pH from 5.9 to 7.4 in the presence of acetate and the alternative electron acceptor nitrate. Compared to normoxia, hypoxia elevated the expression of SPI-1 genes encoding T3SS-1 translocators and effectors, which resulted in higher invasion and effector translocation in epithelial cells. While hypoxia and pH shift only marginally altered the gene expression of SPI-1 regulators, including the SPI-1 repressor *hilE*, hypoxia and pH shift completely incapacitated HilE in a post-translational manner, ultimately promoting SPI-1 activity. From these findings, we conclude that O_2_-dependent HilE function allows for ultrasensitive adaptation of SPI-1 activity in environments with varying O_2_ availability such as the intestinal tract.

## Introduction

**Figure 1:**
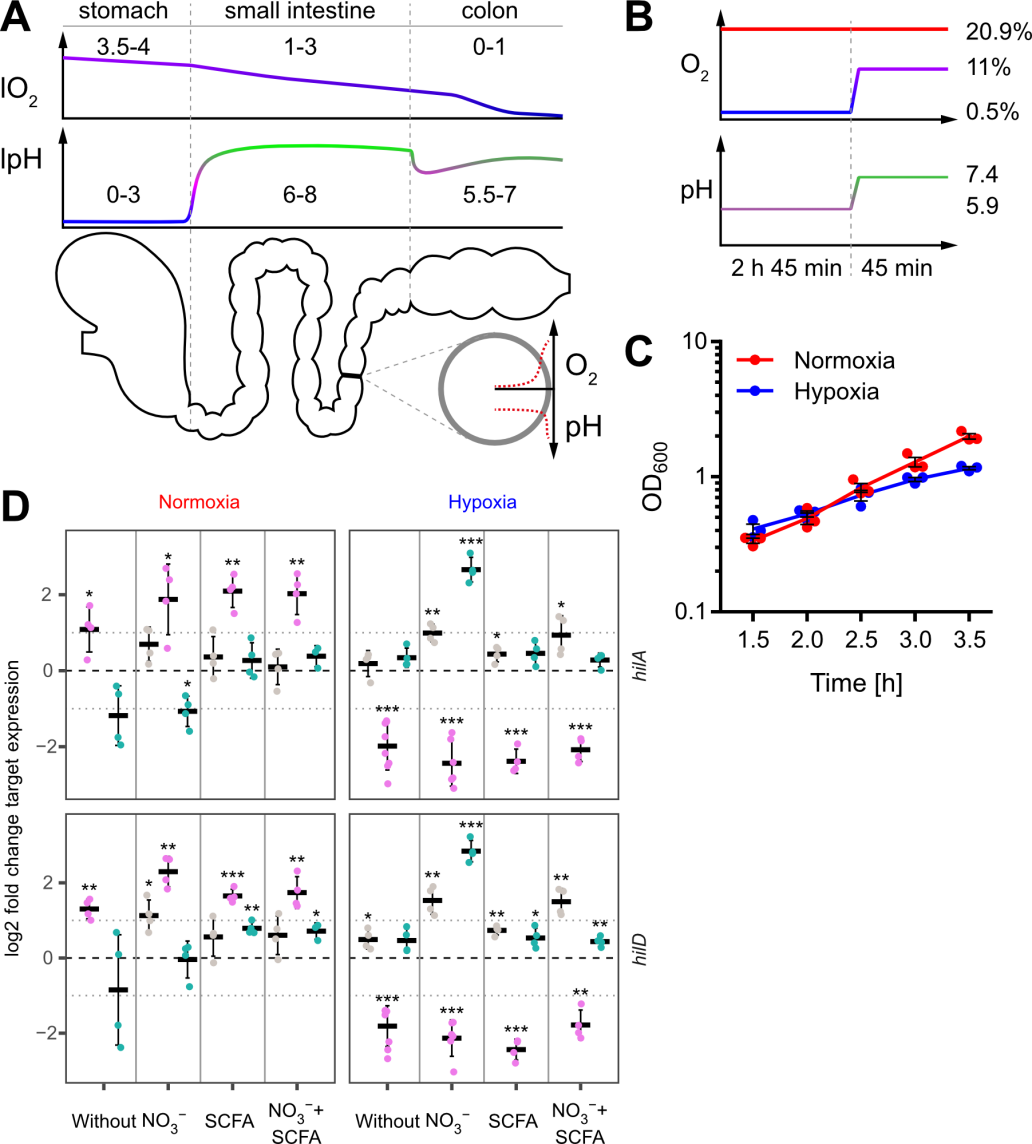
*In vitro* model to study the impact of oxygen on *Salmonella* invasion. **(A)** Proximal to distal changes in luminal O_2_ (lO_2_) and luminal pH (lpH) within the human gastrointestinal tract based on literature data (upper panels). The schematic cross section through the gut illustrates the radial O_2_ and pH gradients overlaying the longitudinal gradients. **(B)** Oxygen and pH applied during STM growth for comparative analyses between normoxia (20.9% O_2_) and hypoxia (two-step O_2_ gradient from 0.5 to 11%) in combination with pH shift (from pH 5.9 to 7.4). **(C)** Growth kinetics of STM WT under normoxia and using the hypoxic conditions shown in **(B)**. Data (n = 3) presented as mean ± SEM. **(D)** Expression of *hilA* and *hilD* were measured by RT-qPCR. STM WT was cultured under normoxia or with a two-step O_2_ gradient (hypoxia) as shown in **(B)**. Media was supplemented as indicated with pH adjusted to 7.4 (grey), 5.9 (purple) or shifted from 5.9 to 7.4 (green). Data shown was normalized to *gyrB* and normoxic samples grown in plain LB w/o pH shift (ΔΔC_t_, dashed line). Depicted are mean ± SD (n=4-6) with statistical significance calculated using a one sample *t*-Test against 0 defined as * for *p* < 0.05, ** for *p* < 0.01 and *** for *p* < 0.001.

With an estimated 153 million cases worldwide, infections with non-typhoidal *Salmonella* (NTS) account for a significant proportion of the burden of disease caused by enteropathogenic bacteria. As a result, *Salmonella* infections cause the highest loss of disability-adjusted life years (DALY) of any foodborne pathogen (Kirk et al., 2015). For successful colonization, enteropathogens such as *Salmonella enterica* subsp*. enterica* serovar Typhimirum (*S*. Typhimurium) have to adapt to hostile host environments. Stomach acids are thought to kill more than 99.99% of orally applied *Salmonella* during transit to the small intestine (Gorden and Small, 1993). After passaging the highly acidic stomach, the intraluminal pH rapidly rises to neutral levels in the ileum followed by a decrease to mildly acidic values in the colon (Evans et al., 1988). Luminal acidic pH is neutralized by bicarbonate in the mucus overlaying the enterocytes generating a steep pH gradient from lumen to gut mucosa (Atuma et al., 2001) (**Figure 1A**).

Low oxygen (O_2_) tension is another environmental cue within the host organism (Jantsch and Schödel, 2015). During its passage through the gastrointestinal system*, Salmonella* faces a proximal to distal ever-decreasing luminal O_2_ concentration (lO_2_). In the naïve gut, lO_2_ decreases from ~4% in the stomach to 1-3% in the small intestine and reaches anaerobiosis in the distal colon (He et al., 1999) (**Figure 1A**). At the same time, a zone of relative oxygenation exists generated by arterial blood in the vicinity of enterocytes (Marteyn et al., 2010). However, pathogen-elicited, neutrophil-driven inflammation of the gut is able to reduce further the availability of O_2_ (Jantsch and Schödel, 2015; Jennewein et al., 2015; Campbell et al., 2019; Tinevez et al., 2019), which impairs antibacterial effector mechanisms against Enterobacteriaceae like *E. coli* (Wiese et al., 2012) or *S*. Typhimurium (Jennewein et al., 2015). Under these pathophysiological conditions, *S.* Typhimurium shows an increased activity of the *Salmonella* pathogenicity island 2 (SPI-2)-encoded type III secretion system (T3SS-2) that together with the blunted antimicrobial host response fosters intracellular replication (Jennewein et al., 2015).

Oxygen and other environmental signals, such as osmolarity, pH, bile acids or long- and short-chain fatty acids (LCFAs/SCFAs), also control the expression and activity of another type III secretion system (T3SS-1), which is encoded by *Salmonella* pathogenicity island 1 (SPI-1) (Eade et al., 2016; Golubeva et al., 2016; Lou et al., 2019). T3SS-1 is critical for efficient bacterial invasion into host cells and for eliciting an inflammatory response that benefits the pathogen to compete with the host microbiota (Stecher et al., 2007). A surprisingly complex regulatory network integrates environmental signals of different origin that eventually merge via HilD in a feed-forward regulatory loop consisting of the transcriptional regulators HilC, HilD and RtsA (Ellermeier et al., 2005; Golubeva et al., 2012; Narm et al., 2020). Each of these proteins can promote transcription of the SPI-1 encoded HilA, which drives expression of the T3SS-1 structural genes and several effectors (Ellermeier et al., 2005).

HilD activity is subject to complex post-transcriptional as well as post-translational regulation. A vastly decreased HilD half-life was demonstrated in the presence of the SCFA propionate and bile acids (Hung et al., 2013; Eade et al., 2016). Recent results suggested that dietary or microbiota-derived bile acids and LCFAs directly inhibit dimerization of the AraC/XylS-type transcription factor HilD and thereby impede its ability to bind to target DNA (Joiner et al., 2023; Yang et al., 2023). In a similar mechanism, the HilD-specific inhibitor HilE binds HilD and thus prevents dimerization (Joiner et al., 2023). Expression of *hilE* depends on the two-component signal transduction system (TCS) PhoPQ via the orphan response regulator FimZ (Baxter and Jones, 2015) and is repressed by Mlc, a global regulator of carbohydrate metabolism (Kimata et al., 1998). On the post-transcriptional level, the small regulatory RNAs (sRNAs) SdsR and Spot 42 increase *hilD* mRNA levels by interfering with RNAse E-dependent mRNA degradation (Abdulla et al., 2023). The sRNA MicC was shown to decrease HilD protein levels through blockage of the *hilD* mRNA ribosome binding site (RBS). Expression of *micC* depends on SlyA, while the TCS OmpR/EnvZ represses its transcription (Cakar et al., 2022). Similarly, the two sRNAs ArcZ and FnrS were shown to decrease HilD protein levels by blocking the RBS of the *hilD* transcript. While transcription of *arcZ* is repressed by the TCS ArcAB (aerobic respiratory control), *fnrS* expression depends on Fnr (fumarate-nitrate reduction) (Kim et al., 2019). Fnr and ArcAB control together the adaptation from aerobic to anaerobic growth in *E. coli* and *Salmonella* (Brown et al., 2022) and, as a result, HilD production is optimal at intermediate O_2_-levels (Kim et al., 2019).Low oxygen does not only serve as a SPI-1 inducing signal but also results in a less efficient generation of energy. When O_2_ is limited or absent, other, less efficient means of energy generation will be utilized with major impact on bacterial metabolism, fitness and virulence (Unden et al., 2002; Jennewein et al., 2015). Successful colonization of the hypoxic gut by Enterobacterales such as *E. coli* or *Salmonella* largely depends on the availability of nitrate (NO_3_^-^) which is the preferred electron acceptor due to its high redox potential (Lopez et al., 2012; Winter et al., 2013; Byndloss et al., 2017). Moreover, NO_3_^-^ is abundant *in vivo* as a product of nitric oxide (NO) oxidation (Winter et al., 2013), the production of which is controlled by PPAR-γ-dependent signaling in intestinal epithelial cells (Byndloss et al., 2017).

In the present study, we established an *in vitro* growth protocol under hypoxic conditions, using medium supplemented not only with NO_3_^-^, but also with acetate. Acetate is the most abundant SCFA *in vivo* within the distal ileum and the caecum (Lawhon et al., 2002). As low O_2_ levels in the lower intestine are accompanied by increased pH *in vivo*, we included a shift in pH as well. Using these refined *in vitro* conditions, we detected a novel, O_2_-dependent posttranslational regulation of SPI-1 function. The repressor HilE failed to inhibit HilD function, which resulted in increased transcriptional activity of SPI-1 genes, T3SS-1 effector translocation and invasion of non-phagocytic cells.

## Results

### *In vitro* growth conditions to assess the impact of oxygen at the early stages of *Salmonella* infection

*Salmonella* is exposed to steadily decreasing O_2_ levels when passaging from the proximal to the distal part of the intestinal tract. However, when *Salmonella* approaches the enterocytes of the small intestine, O_2_ diffusing from arterial blood will lead to a sharp increase of the concentration of O_2_ encountered by *Salmonella* (Marteyn et al., 2010) (**Figure 1A**). To date, no technology exists to assess the role of this microenvironmental factor for *Salmonella* virulence *in vivo* in the different regions of the gut. Therefore, we aimed to develop an *in vitro* growth protocol reflecting these changes. We opted for a sub-culture model until reaching the late logarithmic growth phase because this allows for homogeneous, fast O_2_ equilibration with the surrounding atmosphere. The growth medium was supplemented with acetate to account for the high concentrations of this SCFA in the mammalian intestinal tract (Lawhon et al., 2002). Sub-culturing was started in mildly acidic (pH 5.9) medium supplemented with 100 mM nitrate (NO_3_^-^) at 0.5% O_2_ for 2 h 45 min. To simulate the entry into the partially oxygenated environment of enterocytes, we increased the O_2_ level from 0.5% to 11% (in the following referred to as ‘hypoxia’) during the last 45 minutes of sub-culture (**Figure 1B** upper panel). In parallel, the pH was also elevated from 5.9 to 7.4 mimicking the neutralizing effect of the small intestine and mucus (**Figure 1B** lower panel). Under these growth conditions, bacteria grew slightly slower compared to normoxic controls (**Figure 1C**).

### Impact of growth conditions on SPI-1 transcription

Late logarithmic growth as applied here is a well-characterized method to induce expression of T3SS-1 under normoxia (Ibarra et al., 2010). To elucidate the impact of the chosen *in vitro* conditions on T3SS-1 transcription, we measured *hilA* and *hilD* expression using all combinations of media supplements, oxygen and pH steps by RT-qPCR. As expected (Ellermeier et al., 2005), both genes were expressed in a largely synchronous manner (**Figure 1D**, **Supplementary Figure S1**). While growth in acidified LB (pH 5.9) led to increased expression of both genes under normoxia, hypoxia decreased transcription of *hilA* and *hilD* irrespective of further media supplementation (**Figure 1D**). Interestingly, addition of NO_3_^-^ led to significantly increased *hilAD* expression under O_2_-limiting and pH shift (pH 5.9 to pH 7.4) conditions, but only in the absence of acetate. In contrast, addition of the SCFA acetate alone or together with NO_3_^-^ did not alter or only marginally increased the expression of the two transcription factors, regardless whether the pH was 5.9 or shifted from 5.9 to 7.4 (**Figure 1D**, **Supplementary Figure S1**). In conclusion, we observed for bacteria grown in acidified LB significantly up- and downregulated *hilAD* mRNA levels under normoxic and hypoxic conditions, respectively. The detrimental effect of low pH under hypoxia could be rescued when pH was raised to 7.4 at the end of the incubation. Except when pH shift was combined with hypoxia and NO_3_^-^ supplementation, no major differences were observed in all other media conditions. This included also the combination of SCFA and NO_3_^-^ likely resembling the *in vivo* situation. To elucidate the hypoxia-induced transcriptional changes under these conditions, we continued to analyze the whole transcriptome using RNA-seq.

### Elevated transcription of T3SS-1 ‘late genes’ under hypoxia

**Figure 2:**
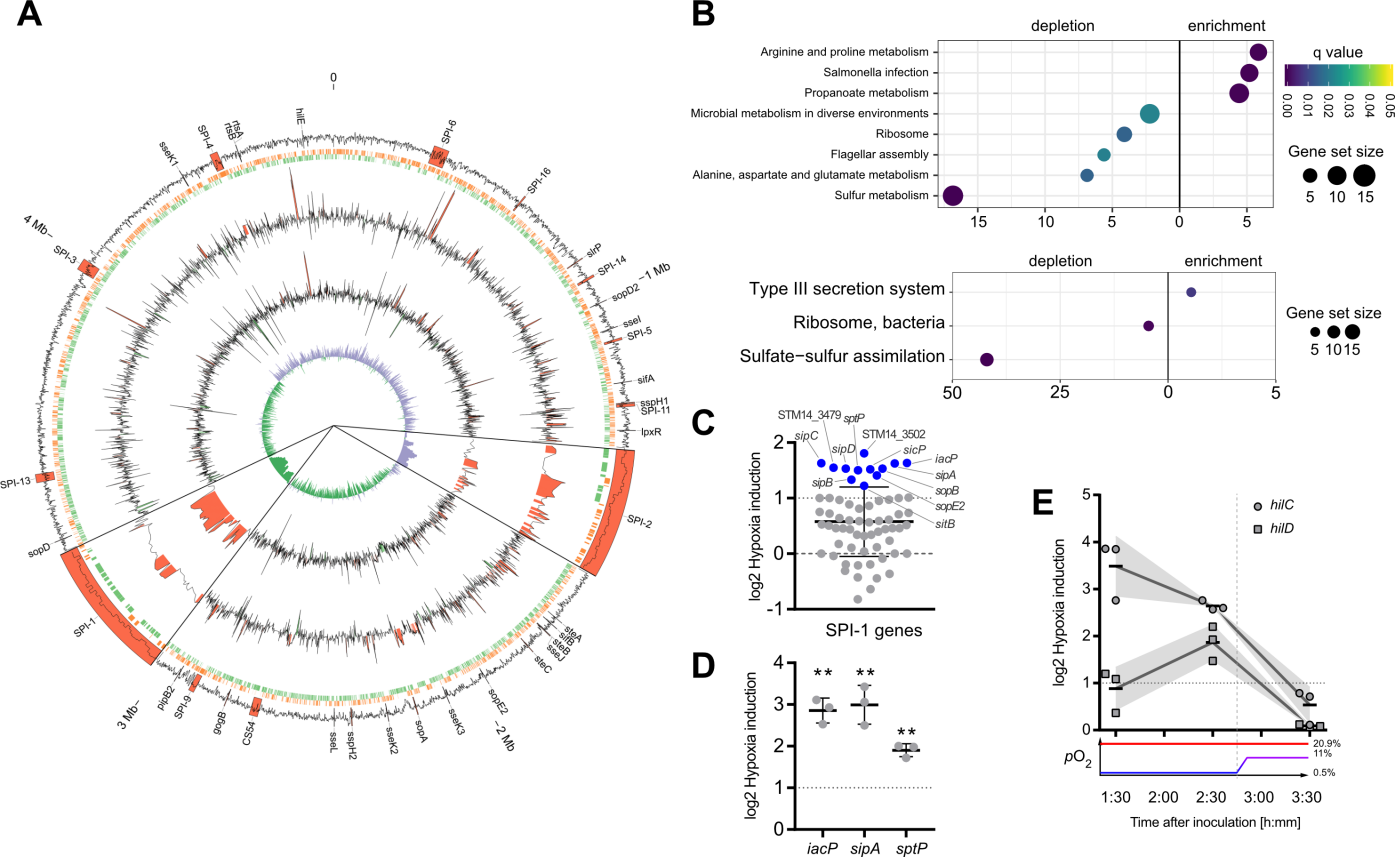
Activation of SPI-1 transcription in an optimized *in vitro* model. **(A)** The data of RNA-seq experiments was mapped to the genome of STM strain 14028s. Tracks from outside to inside are: (1) G/C content with important virulence loci labeled and annotated in red, (2) open reading frames on leading (red) and lagging (green) strand, (3) log2-transformed hypoxia expression normalized to normoxia of STM WT, (4) log2-transformed hypoxia expression normalized to normoxia of STM with HilE expression induced from pHilE, (5) G/C skew. Changes >2-fold are highlighted in red (positive) or green (negative). SPI-1 and SPI-2 loci are shown 10x magnified. **(B)** Identification of significantly enriched KEGG pathways (upper panel) and KEGG modules (lower panel) on the basis of genes with more than 2-fold changed expression under hypoxia. **(C)** Impact of hypoxia on the expression of SPI-1 encoded or -associated (regulators, effectors) genes. Genes with more than 2-fold increased expression are shown in blue. **(D, E)** Expression of *iacP*, *sipA* and *sptP* after 3.5 h **(D)** or kinetic of *hilC* and *hilD* expression **(E)** based on RT-qPCR. Hypoxia expression was normalized to that of *gyrB* and subsequently to normoxic controls (ΔΔC_t_). The O_2_ concentrations applied throughout the kinetic are shown below. Depicted are mean ± SD (n=3) with statistical significance calculated using a one sample *t*-Test against 0 defined as ** for *p* < 0.01.

In order to gain further insights into the impact of hypoxia on *Salmonella* virulence gene expression, we analyzed the whole transcriptome using RNA-seq. Samples for RNA-seq were collected from cultures that were supplemented with NO_3_^-^ and acetate after pH shift and either grown under ambient air (“normoxia”) or after O_2_ shift (0.5% to 11%; “hypoxia”) during sub-culture. In line with previous observations (Lee and Falkow, 1990; Jones and Falkow, 1994; Jiang et al., 2017), we observed an increased expression of T3SS-1 under hypoxia (**Figure 2A**). Interestingly, we also found a robust hypoxic induction of the SPI-2 encoded T3SS-2 (**Figure 2A**). Slightly acidic pH (Löber et al., 2006) and low O_2_ (Jennewein et al., 2015) are known to facilitate SPI-2 expression. Our results are in accordance with previous observations that co-induction of both T3SS systems *in vivo* contribute to epithelial traversal (Müller et al., 2012) and enterocolitis (Coburn et al., 2005). Bioinformatic analysis of the genes that were more than 2-fold induced by hypoxia revealed a significant enrichment of the ‘arginine and proline metabolism’, ‘propanoate metabolism’ and ‘*Salmonella* infection’ Kyoto Encyclopedia of Genes and Genomes (KEGG) pathways (**Figure 2B**, upper panel). In line with that, analysis of KEGG modules in the same gene set showed a highly significant enrichment of the ‘Type III secretion system’ module (**Figure 2B**, lower panel). Furthermore, as described before (Ibarra et al., 2010), our data showed also upregulation of virulence-associated type I fimbriae when O_2_ was restricted (**Supplementary Dataset S1**). When looking at genes downregulated at least 2-fold under hypoxia, several other KEGG pathways were enriched, e.g. ‘ribosomes’, ‘flagellar assembly’ and ‘sulfur metabolism’ (**Figure 2B**), as well as the modules for ‘ribosome, bacteria’ and ‘sulfate-sulfur assimilation’ (**Figure 2B**). A closer look on the SPI-1 regulon genes revealed that the majority (50 genes) of 64 genes was upregulated under hypoxic conditions, with 12 genes exhibiting a more than 2-fold increase in mRNA levels (**Figure 2C**) (**Supplementary Dataset S1**). Interestingly, seven of these 12 genes encode for later substrates of the T3SS-1, i.e. translocators (SipBCD) or effectors (SipA, SopBE2, SptP). SicP, the specific chaperone of the effector SptP (Stebbins and Galán, 2001) was also found in this group as well as IacP. IacP is homolog to acyl carrier proteins (Viala et al., 2013) and was shown to acylate SipB, thereby promoting insertion of the translocator into the host cell membrane (Viala et al., 2017). Upregulation was exemplarily confirmed by RT-qPCR experiments for *iacP*, *sipA* and *sptP* genes (**Figure 2D**). In summary, we found that hypoxia had a major impact on *Salmonella* transcription including a vast set of virulence genes. In our experimental setting genes of both T3SSs were upregulated significantly. With regard to T3SS-1, a subset of genes known to be involved in later stages of the assembly and translocation process were induced. In a next step we wanted to elucidate the origin of this induction by analyzing expression of the SPI-1 transcription factors HilC and HilD over time.

### Early hypoxia-dependent induction of HilC and HilD regulators

In line with our qPCR data (**Figure 1D**), RNA-seq data obtained 3.5 h after hypoxic exposure did not reveal significant upregulation of the SPI-1 transcriptional activator HilD. Together with HilC, HilD is at top hierarchical position in the SPI-1 regulatory cascade (Ellermeier et al., 2005). Therefore, we speculated that expression of these regulators might have been induced earlier upon exposure to hypoxic conditions. To address this hypothesis, transcription of *hilC* and *hilD* was quantified by RT-qPCR in *Salmonella* grown at low O_2_ (0.5%) for 1.5 h and 2.5 h and after shifting to 11% O_2_ at 3.5 h. Compared to normoxic controls, *hilC* expression was highest already after 1.5 h under hypoxia and steadily declined thereafter. Expression of *hilD* transcripts followed a different kinetic with maximum expression after 2.5 h under hypoxia (**Figure 2E**). Hypoxic induction of *hilD* mRNA was lower than that of *hilC* at any time point with both reaching their lowest level at 3.5 h (**Figure 2E**). These observations indicate that as early as 1.5 h after exposure to hypoxia expression of key T3SS-1 transcription factors is induced. This is the foundation of T3SS-1 ‘late’ gene expression observed in RNA-seq after 3.5 h. Next, we wanted to test whether hypoxia-induced expression of T3SS-1 resulted also in elevated *Salmonella* invasion.

### Low O_2_ fosters *Salmonella* invasion

**Figure 3:**
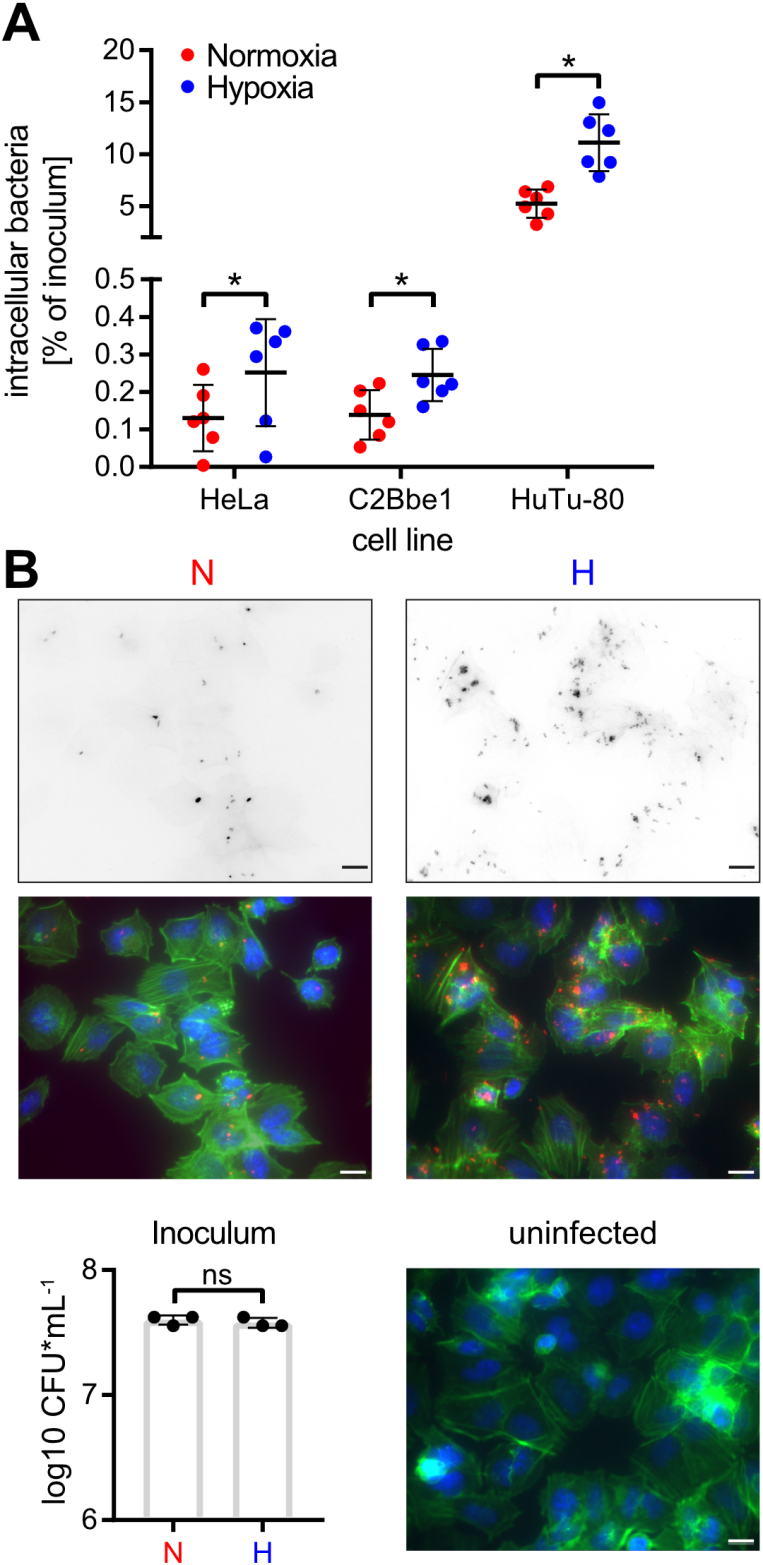
Hypoxia promotes *Salmonella* invasion. **(A)** Invasion of STM WT grown under normoxic or hypoxic conditions in the indicated cell lines from six independent experiments is depicted. Statistical significance was calculated comparing normoxic and hypoxic conditions as indicated using one-way ANOVA with Holm-Šídák multiple comparisons test and was defined as * for *p* < 0.05. **(B)** Fluorescence microscopy images of HuTu-80 cells infected with normoxically (N) or hypoxically (H) grown STM WT expressing mCherry. Shown are the red fluorescence channels (bacteria, upper panels), the combined fluorescence channels with bacteria in red, F-actin in green and nuclei in blue (middle panels) and quantification of the inoculi used for infection (lower left panel) and an uninfected control (lower right panel). Scale bar = 10 µm.

To elucidate the impact of hypoxia on *Salmonella* invasion, the three cell lines HeLa (human epitheloid cervix carcinoma), C2BBe1 (human enterocytes) and HuTu-80 (duodenal adenocarcinoma) were infected with normoxically or hypoxically grown bacteria. Compared to normoxic controls, a significantly higher proportion of the inoculum of hypoxically grown *Salmonella* was able to enter the host cells as quantified by gentamicin protection assays (**Figure 3A**). Likewise, we detected more RFP-expressing intracellular bacteria after hypoxic growth within HuTu-80 cells by fluorescence microscopy (**Figure 3B**). We used the HuTu-80 infection model to elucidate the impact of the different media supplements without or combined with pH and O_2_ shifts. Addition of acetate strongly promoted invasion by wildtype (WT) STM under hypoxia with or without pH shift (**Supplementary Figure S2**, **Figure 3A**). While there was no significant difference in invasiveness between normoxically and hypoxically grown bacteria using neutral LB medium with or without NO_3_^-^, a pH shift in the same media strongly attenuated invasion of hypoxic bacteria (**Supplementary Figure S2**). In summary, hypoxia is able to promote *Salmonella* invasion in the presence of the SCFA acetate. As shown earlier these conditions induce T3SS-1 expression and in the next experiments we wanted to test, whether this also results in higher bacterial effector translocation.

### Increased T3SS-1 activity under hypoxia

**Figure 4:**
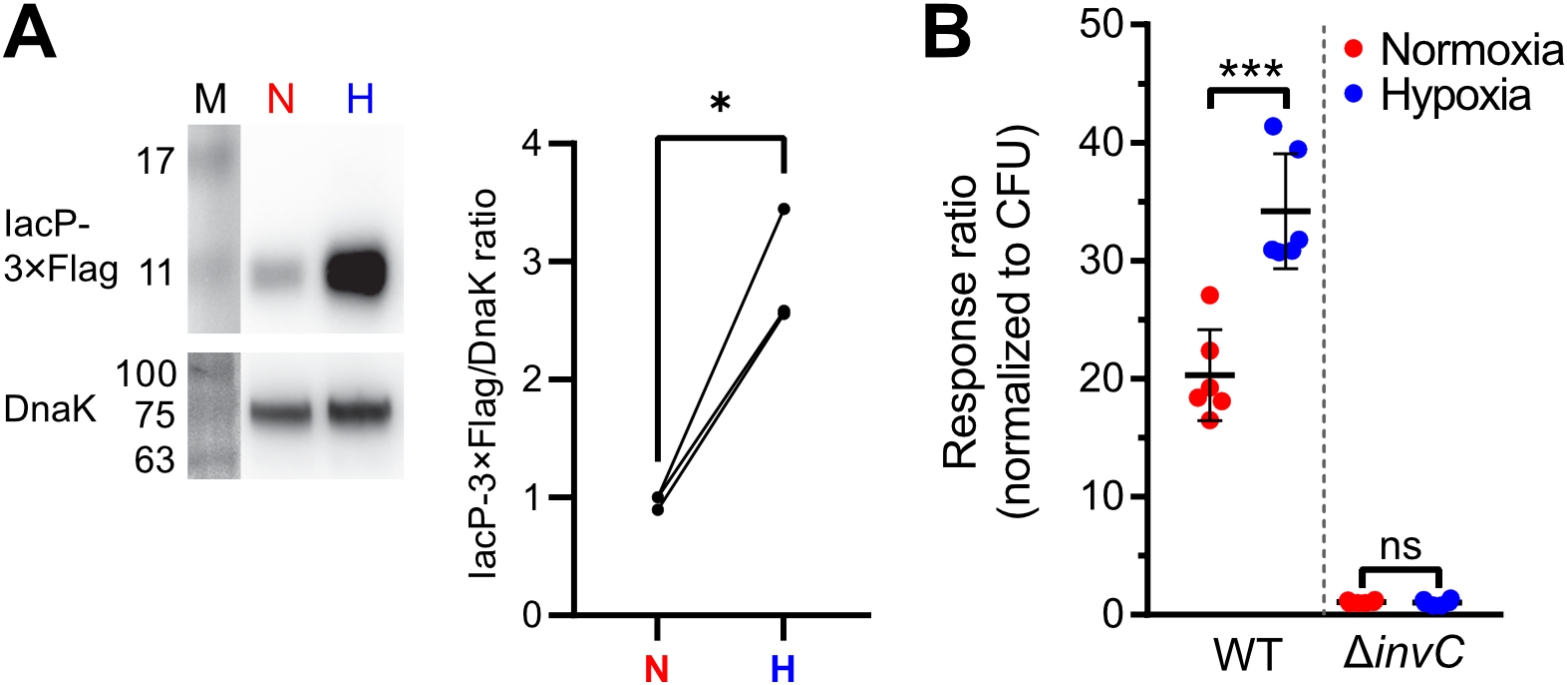
Increased T3SS-1 expression and activity under low O_2_. **(A)** Expression of chromosomally 3xFlag-tagged IacP after normoxic (N) or hypoxic (H) growth as detected with an anti-3xFlag antibody (upper panel). To test for equal loading, samples were probed with an anti-DnaK antibody (lower panel). M, molecular weight marker with band sizes as indicated. On the right, ratiometric quantification of band intensities for three independent experiments with statistical analysis using paired *t*-test with * defined as *p* < 0.05 is shown **(B)** Translocation of a chromosomally encoded SipA-BlaM fusion protein in HeLa cells. The response ratio as a measure of effector translocation was calculated after ratiometric detection of the cleaved and intact BlaM substrate CCF2-AM and normalization to bacterial numbers (CFU). A mutant with a non-functional T3SS-1 (*invC*) was included as a negative control. Data of six independent experiments done in duplicates are shown and statistical significance was calculated using unpaired, two-tailed Students *t*-test and was defined as ns for *p* > 0.05 and *** for *p* < 0.001.

Invasion of non-phagocytic cells but also triggering (and subsequent restriction) of an inflammatory response highly depends on the translocation of effectors through the T3SS-1 (Galán, 2021). To test, whether our hypoxic growth conditions also result in increased SPI-1 effector levels, we employed an epitope-tagged version of IacP as a reporter. Expression of IacP-3×Flag was elevated under hypoxia, while the amounts of the cytoplasmic chaperone DnaK remained constant under both conditions (**Figure 4A**). Next, we tested whether enhanced SPI-1 transcription and effector protein levels also amount to increased effector translocation into host cells. The translocation of the T3SS-1 effector SipA was quantified using a Foerster resonance energy transfer (FRET)-based reporter assay (Schlumberger et al., 2007). Hypoxically grown *Salmonella* translocated significantly more SipA in host cells than normoxic controls (**Figure 4B**). No effector translocation was observed in a Δ*invC* mutant lacking a functional T3SS-1 (**Figure 4B**). These results demonstrate that an *in vitro* growth condition combining a pH shift from slightly acidic to neutral with a hypoxic atmosphere was able to enhance T3SS-1 protein expression and effector translocation, which eventually led to higher invasion of non-phagocytic cells.

### Hypoxic shifts *hilD* bimodal expression and possible role of HilE

**Figure 5:**
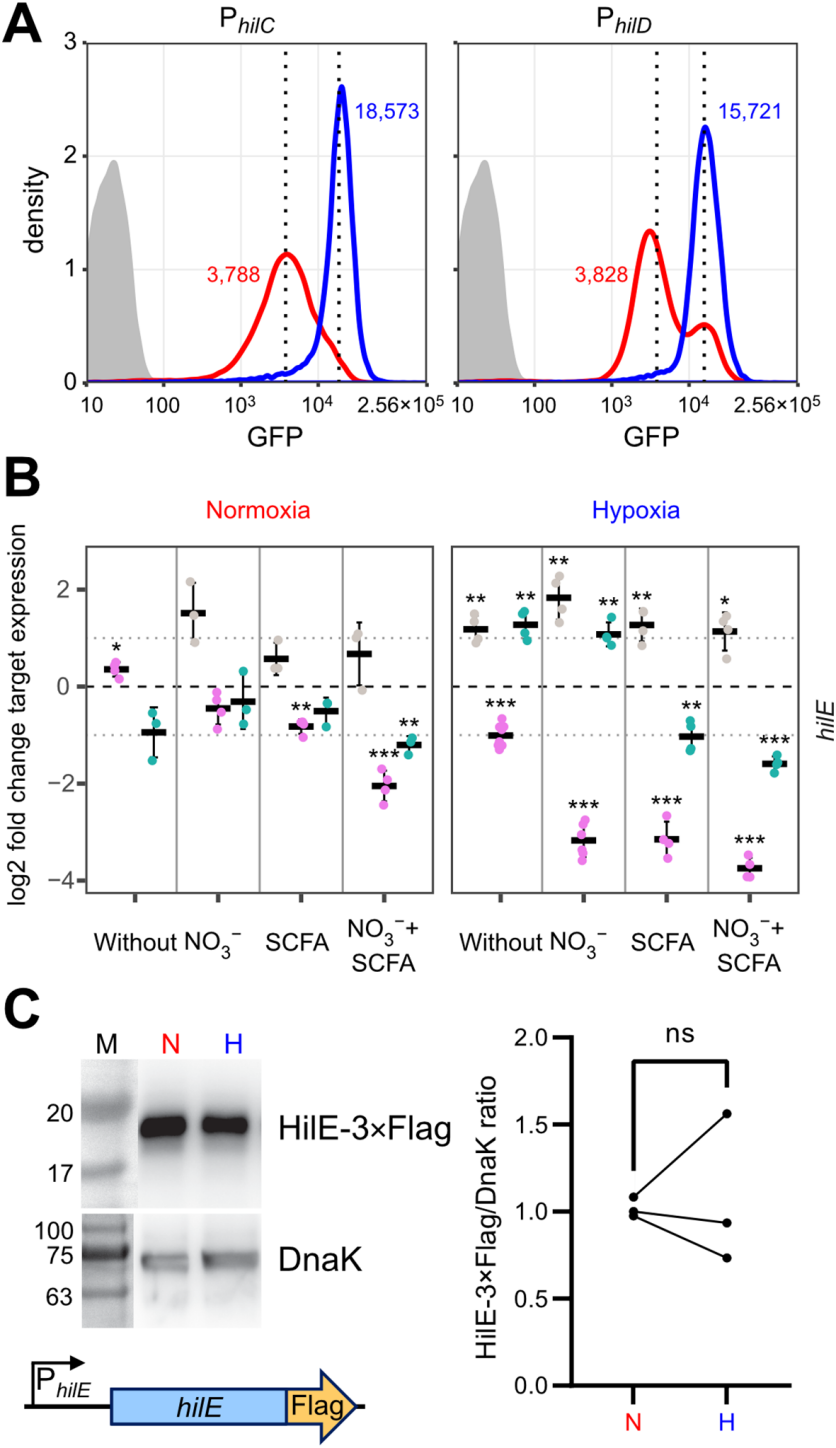
O_2_-dependent, population-scale regulation of T3SS-1 transcription factors and expression of *hilE*. **(A)** Flow cytometric analysis of STM WT without plasmid (grey) or harboring GFP reporter fusions with promoters of *hilC* (P*_hilC_*) and *hilD* (P*_hilD_*) after 3.5 h of normoxic (red) or hypoxic (blue) growth. Dotted lines and numbers indicate median fluorescence intensity. Data shows one representative out of 10 similar experiments. **(B)** Expression of *hilE* was measured by RT-qPCR. STM WT was cultured under normoxia or with a two-step O_2_ gradient (hypoxia). Media was supplemented as indicated with pH adjusted to 7.4 (grey), 5.9 (purple) or shifted from 5.9 to 7.4 (green). Data shown was normalized to *gyrB* and normoxic samples grown in plain LB pH 7.4 (ΔΔC_t_, dashed line). Depicted are mean ± SD (n=3) with statistical significance calculated using a one sample *t*-Test against 0 defined as * for *p* < 0.05, ** for *p* < 0.01 and *** for *p* < 0.001. **(C)** On the left expression of plasmid-encoded HilE-3×Flag from its natural promoter (schematic representation below) after normoxic (N) and hypoxic (H) growth of STM WT probed with an anti-Flag antibody. Equal sample loading was probed with an anti-DnaK antibody. M, molecular weight marker with band sizes as indicated. On the right, ratiometric quantification of band intensities for three independent experiments with statistical analysis using paired *t*-test is shown (ns, not significant).

How can environmental cues present in our hypoxic *in vitro* model result in higher T3SS-1 expression and activity? Environmental signals are thought to be integrated via HilD in the HilC-HilD-RtsA feed-forward loop of transcriptional regulators (Ellermeier et al., 2005). As another regulatory layer bistability of T3SS-1 expression has been described (Bailly-Bechet et al., 2011; Sturm et al., 2011; Figueroa-Bossi et al., 2022). To gain more insight in the differential regulation of *hilC* and *hilD* within the bacterial population, we constructed transcriptional reporter fusions of the promoters of *hilC* (P*_hilC_*), *hilD* (P*_hilD_*) and a destabilized variant of superfolder GFP (sfGFP). In contrast to the RT-qPCR data, a clear hypoxia-dependent induction of both reporters was evident at the 3.5 h time-point (**Figure 5A**). This difference in kinetics could be explained with translation and maturation of the sfGFP reporter protein of the fluorescence reporter construct. Similar to the RT-qPCR results, P*_hilC_* reporter activity was again higher than P*_hilD_*-based fluorescence. In line with earlier studies (Temme et al., 2008; Clark et al., 2011), we found a bimodal expression of *hilD* under normoxic conditions. Interestingly, the P*_hilD_* reporter switched from a bimodal to a monomodal expression pattern when exposed to hypoxia (**Figure 5A**). Because HilE was shown to influence the size of the bimodal fractions within a population (Sturm et al., 2011; Diard et al., 2013), we speculated about a role of this SPI-1 repressor for the observed phenotype. Mechanistically, HilE inhibits HilD post-translationally through direct interaction, thereby interfering with the HilD DNA binding interface (Baxter et al., 2003; Grenz et al., 2018). To test, whether decreased hypoxic expression of *hilE* could play a role, we measured expression of *hilE* by RT-qPCR using all combinations of media supplements, oxygen and pH steps. Significantly lower amounts of *hilE* transcripts were detected in the presence of SCFA when combined with pH shift and normoxia, 0.5% O_2_ or with the two-step oxygen gradient (**Figures 5B**, **Supplementary Figure S3**). HilE transcription was strongly reduced after growth in pH 5.9 media irrespective of the media supplement. This effect was especially pronounced when combined with hypoxia (**Figure 5B**). All culture conditions lacking acetate and/or the pH shift showed increased *hilE* expression when O_2_ was shifted from 0.5 to 11% (**Figure 5B**). However, compared with normoxic controls, HilE protein levels were not significantly different under hypoxic growth conditions using an epitope-tagged protein (**Figure 5C**). Because reduced transcription after 3.5 h growth did not result in diminished HilE protein levels, while *hilD* expression is shifted to monomodal, we reasoned that the hypoxic environment might interfere with HilE function itself.

### Hypoxia inhibits HilE function

**Figure 6:**
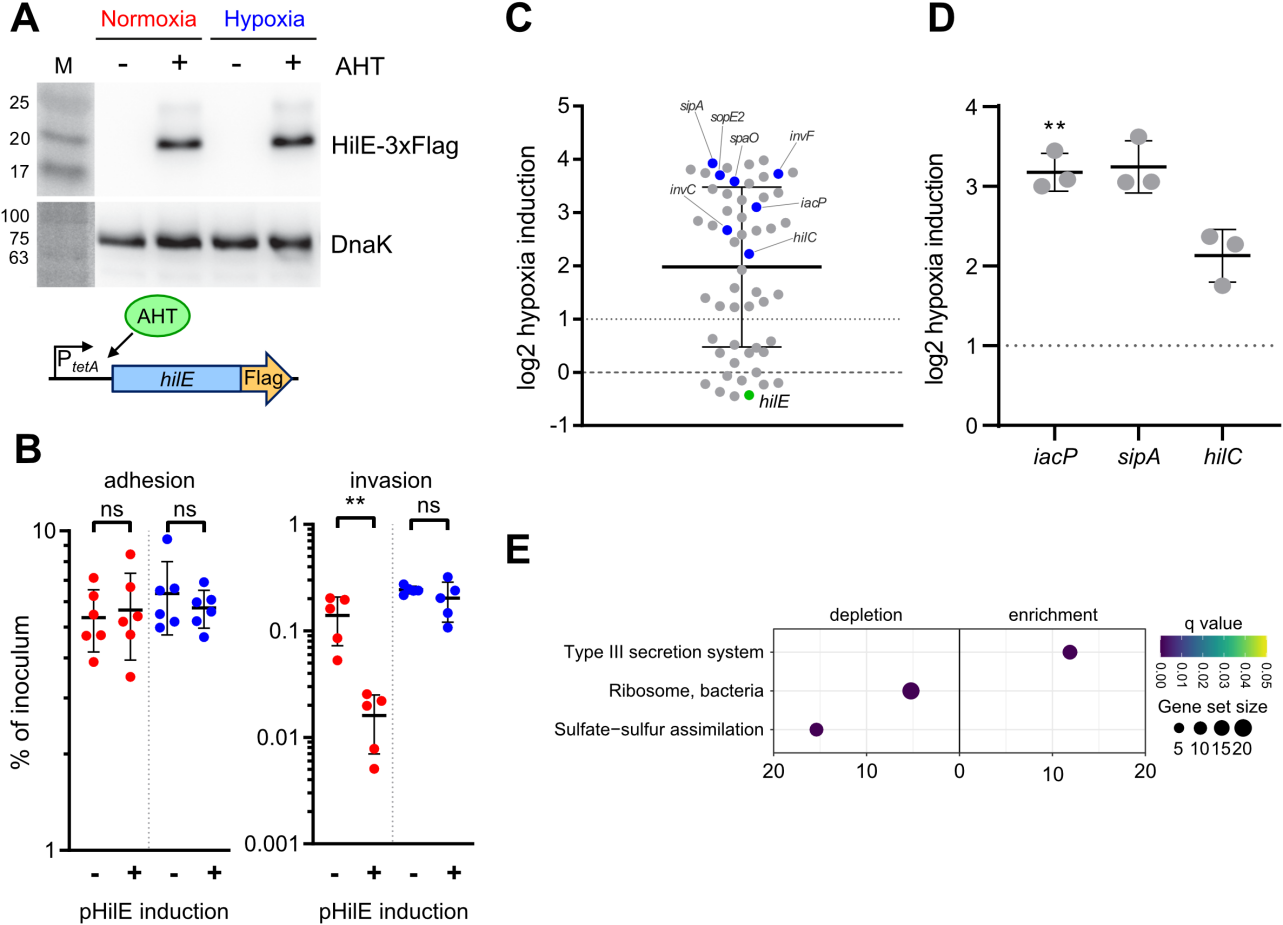
O_2_-dependent HilE function. **(A)** Expression of plasmid-encoded HilE-3×Flag from a tetracycline-inducible promoter (schematic representation of the construction below) after normoxic and hypoxic growth of STM WT with or w/o addition of 50 ng/µL anhydrotetracycline (AHT) as detected with an anti-3×Flag antibody. Equal sample loading was probed with an anti-DnaK antibody. M, molecular weight marker with band sizes as indicated. **(B)** C2Bbe1 cells were infected with STM WT harboring pHilE for tetracycline-inducible expression of HilE. Bacteria were either grown under norm- or hypoxia and with or w/o addition of the inducer AHT as indicated. The left panel shows adherent bacteria while invasion was quantified in the right panel. Data of five or six independent experiments done in duplicates are shown and statistical significance was calculated using unpaired, two-tailed Students *t*-test and was defined as ** for *p* < 0.01, ns, not significant. In **(C)** RNA-seq-based differential expression of SPI-1 encoded or associated (regulators, effectors) genes is depicted. Hypoxia expression was normalized to normoxic controls. Expression of *hilE* was induced in normoxic and hypoxic samples with addition of 50 ng/mL AHT as shown in **(A)**. Genes tested with RT-qPCR **(D)** or encoding for major regulators are shown in blue and *hilE* is marked in green. **(D)** Expression of *iacP*, *sipA* and *hilC* based on RT-qPCR. Hypoxia expression was normalized to that of *gyrB* and subsequently to normoxic controls. Statistical significance of data from three independent experiments done in duplicates was calculated using a one sample *t*-Test against 0 (no regulation) as theoretical mean and was defined as ** for *p* < 0.01. **(E)** Identification of significantly differential abundant KEGG modules in strains expressing *hilE* on the basis of genes with more than 2-fold changed expression under hypoxia.

To address a possible effect of hypoxic growth on the ability of HilE to inhibit T3SS-1 function, we used a model allowing for tetracycline-inducible expression of HilE. Induction of the system led to the production of equal amounts of HilE under low and ambient O_2_ (**Figure 6A**). When C2BBe1 cells were infected with *Salmonella* harboring the *hilE* expression plasmid under normoxic conditions, bacterial invasion was strongly attenuated upon HilE induction, which is in accordance with its function as a repressor of SPI-1 expression (Baxter et al., 2003). Surprisingly, no significant reduction of intracellular *Salmonella* was evident upon induction of HilE under low O_2_ (**Figure 6B**, left panel). However, bacterial adhesion remained unaffected by HilE expression and O_2_ concentration (**Figure 6B**, right panel). In line with this, RNA-seq of HilE-induced bacteria demonstrated that hypoxia increased markedly the transcription of 38 SPI-1 genes, including the regulators HilC and InvF (**Figures 6C**, **2A** track No. 2). This strong relative upregulation of SPI-1 regulon genes was confirmed for selected targets by RT-qPCR (**Figure 6D**). The lack of significant differences in *hilE* transcription upon addition of anhydrotetracycline (AHT) in RNA-seq (**Figure 6C**, green dot) corresponded to similar HilE protein levels after induction under hypoxic and normoxic conditions (**Figure 6A**). A KEGG module enrichment analysis identified similar gene sets as with WT STM (**Figure 2B**), but with even higher enrichment of the ‘type III secretion system’ module within enriched gene sets (**Figure 6E**). However, overexpression of the repressor HilE reinforced the observed differences in *Salmonella* invasion between cells grown under low and ambient O_2_. While expression of *hilE* under normoxic growth repressed T3SS-1 transcription and activity as described before (Baxter et al., 2003; Joiner et al., 2023), HilE function was completely abolished when using a hypoxic growth protocol mimicking key environmental cues of the intestinal tract. Thus, this data argue for a significant role for HilE in the O_2_-dependent regulation of *Salmonella* invasion.

## Discussion

For successful gut colonization, *Salmonella* has to compete with the microbiota for scarce nutrients and energy sources. Constant adaptation to changing environmental conditions is required to ensure sufficient energization of metabolism and virulence functions. Flagella-mediated cellular motility and type III secretion are central for *Salmonella* pathogenesis and do require substantial amounts of energy as ATP and proton-motive force (Sturm et al., 2011; Diepold and Armitage, 2015; Saleh et al., 2023). When O_2_ is limited as a terminal electron acceptor in respiration, alternative electron acceptors with markedly reduced energy yields such as NO_3_^-^ (Winter et al., 2013; Shelton et al., 2022), dimethyl sulfoxide (DMSO) (Antunes et al., 2010), fumarate, together with H_2_ as electron donor (Escalante-Semerena and Roth, 1987; Maier et al., 2013; Nguyen et al., 2020), or tetrathionate (Price-Carter et al., 2001; Winter et al., 2010) are utilized. In contrast to other studies (Lee and Falkow, 1990; Russell et al., 2004; Jiang et al., 2017; Kim et al., 2019), we elucidated the role of limited oxygen availability for SPI-1 activity in the presence of the alternative electron acceptor NO_3_^-^ and the SCFA acetate. Moreover, we mimicked the zone of relative oxygenation (Marteyn et al., 2010), which is present around epithelial cells in the gastrointestinal tract.

These differences are likely the reason, why our results on the impact of O_2_ availability on SPI-1 activation differ from some previous findings. Others had published that transcription of the master regulator HilD is controlled by the SPI-14-encoded transcription factor LoiA, whose expression is induced under hypoxia in an ArcAB-dependent manner (Jiang et al., 2017). However, in our setting, we detected only a 1.9-fold increased *loiA* transcription after exposure to hypoxia (**Supplementary Dataset S1**). Likewise, we did not observe any differences in the expression of the small RNAs *fnrS* and *arcZ*, (**Supplementary Dataset S1**) that were implicated in O_2_-dependent post-transcriptional regulation of *hilD* expression (Kim et al., 2019).

Energetic aspects are only one reason, why it is essential for *Salmonella* to apply sophisticated mechanisms for precise control of the expression and activity of T3SS-1. Structural components of T3SSs and flagella are recognized by the NAIP-NLRC4 inflammasome. Upon recognition, this complex activates caspase-1, which, in turn, triggers interleukin (IL)-1β and IL-18 maturation and release that ultimately leads to macrophage pyroptosis (Kieser and Kagan, 2017; Reyes Ruiz et al., 2017) or enterocyte expulsion (Sellin et al., 2014). The resulting inflammatory response is detrimental for the pathogen, but intestinal inflammation also propagates a dysbalanced microbiota that further contributes to the weakened intestinal barrier function (Stecher et al., 2007; Winter et al., 2013). The *Salmonella* population is thought to circumvent the first while profiting from the last effects using cooperative virulence where only a subpopulation of the pathogen expresses T3SS-1 (Ackermann et al., 2008; Sturm et al., 2011). Bimodal activation has been shown for several SPI-1 associated promoters, e.g. P*_prgH_* and P*_sicA_* (Temme et al., 2008; Clark et al., 2011; Sturm et al., 2011). The HilD-inhibiting protein HilE is required for the balancing and evolutionary stabilization of bistable T3SS-1 expression *in vivo* (Diard et al., 2013). Bistable T3SS-1 allows for efficient tissue invasion and ultimately supports luminal *Salmonella* expansion in a mouse colitis model (Diard et al., 2013).

Of note, the studies that demonstrated that the bistable expression of the T3SS-1 expression is of benefit, relied on experimental mouse models in which streptomycin-pretreatment was used (Diard et al., 2013; Arnoldini et al., 2014; Diard et al., 2014). Antibiotic pretreatment is critically required to induce a disease in mice that resembles *Salmonella* gastroenteritis in humans (Barthel et al., 2003). This antibiotic treatment, however, is known to generate an increase availability of luminal oxygen and ultimately facilitates aerobic expansion of *Salmonella* (Rivera-Chávez et al., 2016). Therefore, our *in vitro* model does not really resemble the situation found in streptomycin-pretreated mice. It rather imitates the setting where the microbiota of a host challenged with *Salmonella* is undisturbed. Under these conditions, *S*. Typhimurium induces in mice an infection that rather resembles typhoid fever (Santos et al., 2001). As oral infections of mice with intact microbiota reduce the likelihood that a single *Salmonella* bacterium enters an epithelial cell, our data suggest that low oxygen is an environmental cue which optimizes the ability of *Salmonella* for epithelial cell invasion and subsequently increases the probability to establish an infection in this natural infection situation.

When *Salmonella* were exposed to hypoxia, we observed an increase in *Salmonella* invasion, which was paralleled by a shift from a bimodal expression pattern of the P*_hilD_*::*gfp* transcriptional reporter to an elevated monomodal expression. Similar to bacteria deficient for *hilE* (Sturm et al., 2011; Diard et al., 2013), the population size of cells expressing T3SS-1 is enlarged under hypoxic conditions. When examining HilE function of bacteria exposed to low O_2_, we noted a loss of its inhibitory effect on T3SS-1 activity. A novel post-translational mechanism of HilE regulation became evident, because the levels of HilE protein remained unchanged under hypoxic versus normoxic conditions. Hypoxia-mediated incapacitation of HilE could rapidly foster HilD-dependent enterocyte invasion through T3SS-1.

Our findings offer new insights into the O_2_-dependent virulence regulation in *Salmonella*. In addition to Fnr- and ArcAB-mediated virulence regulation (Fink et al., 2007; Lim et al., 2013; Jiang et al., 2017), our data adds another layer to the complex O_2_-dependent regulation of T3SS-1 expression. We propose a model where under hypoxia the HilD inhibitor HilE is inactive. This allows for an ultrasensitive regulation of SPI-1 activity in response to low O_2_ environments in a post-translational manner. Interestingly, metazoans rely on a post-translational process for O_2_ sensing to ensure rapid responses and adaptation of cellular functions to low O_2_ environments as well. In metazoans, for instance, the stability of the global regulator hypoxia-inducible factor α is rapidly controlled by reversible hydroxylation of two prolyl residues (Kaelin and Ratcliffe, 2008). This suggests that post-translational modification of proteins is a general mechanism employed by organisms for the adaptation of cellular function across different kingdoms of life.

## Materials and methods

### Bacterial strains and culture

Bacterial strains used in this study are listed in **Table 1**. C-terminal epitope tagging of IacP was done by λ Red-mediated recombination as described before (Hoffmann et al., 2017). Briefly, an I-SceI + kanamycin cassette from pWRG717 was amplified with primers IacP-C-scarless-for/rev (**Supplementary Table S1**) and integrated within the STM NCTC 12023 genome yielding WRG513. To generate WRG514, the resistance cassette was removed from WRG513 by a second λ Red recombination step with a PCR product containing the 3×Flag sequence amplified from pWRG447 (Wille et al., 2014) using primers IacP-3xFlag-for/rev (**Supplementary Table S1**).

**Table 1:** Strains used in this study.

Strain	Relevant characteristic(s)	Source or Reference
NCTC 12023	Wild-type, Nal^s^, isogenic to ATCC 14028	NCTC, Colindale, UK
MvP818	Δ*invC* FRT	(Gerlach et al., 2008)
MvP1281	*sipA*::M45::*tem1*	(Hölzer et al., 2009)
MvP1282	Δ*invC* FRT *sipA*::M45::*tem1*	(Hölzer et al., 2009)
WRG513	*iacP*::I-SceI *aph*, Km^r^	This study
WRG514	*iacP*::3×Flag, basis: WRG513	This study

Bacteria were grown O/N at 37°C under normoxia in neutral LB supplemented with appropriate antibiotics. Cultures were adjusted to an OD_600_ of 0.15 in 3 mL either in neutral or acidified (pH5.9) LB, optionally supplemented with 100 mM KNO_3_ (Sigma-Aldrich) and 30 mM sodium acetate (NaOAc, Sigma-Aldrich). Media were equilibrated previously to normoxia (20.9% O_2_) or hypoxia (0.5% O_2_). Cultures were incubated in roller drums under normoxia or within stirred flasks in a hypoxic workstation (Whitley H35 Hypoxystation, Meintrup DWS, Herzlake, Germany) set to 0.5% O_2_. After 2h 45 min. pH of acidified LB was raised to 7.4 with addition of 28 µL 1N NaOH and for hypoxic samples O_2_ concentration was shifted from 0.5% to 11%. Bacterial growth was continued for another 45 min. For selection purposes LB media was supplemented with 50 µg/mL carbenicillin (Cb) (Carl Roth, Mannheim, Germany), 25 µg/mL kanamycin (Km) (Carl Roth), 10 µg/mL chloramphenicol (Cm) (Carl Roth), or 100 ng/mL anhydrotetracycline (AHT) (Sigma-Aldrich, Schnelldorf, Germany) if required.

### PCR and cloning

**Table 2** gives an overview of all the plasmids used in this study which were all constructed by assembly cloning of PCR fragments (Gibson et al., 2009). All primers (Integrated DNA Technology, Munich, Germany) applied in the PCRs are listed in **Supplementary Table S1**. For construction of the GFP reporter plasmids, the vector backbone of pMW82 (Bumann and Valdivia, 2007) was amplified with primers pMW82-Gbs-for and -rev. Primers PhilC-RBS179k-sfgfp-Gbs-for and LVA-pL-Gbs-rev were used to amplify a destabilized variant (Andersen et al., 1998) of superfolder GFP (SFGFP) from template pWRG218 (Jennewein et al., 2015) together with an optimized ribosome binding site (Salis et al., 2009). The *hilC* promoter (P*_hilC_*) was amplified from chromosomal DNA of STM utilizing primers PhilC-pL-Gbs-for and PhilC-RBS179k-sfgfp-Gbs-rev. Assembly of the fragments resulted in pWRG805. Plasmid pWRG806 was constructed in a similar way with primers PhilD-pL-Gbs-for and PhilD-RBS46k-sfgfp-Gbs-rev to amplify the *hilD* promoter (P*_hilD_*) and using PhilD-RBS46k-sfgfp-Gbs-for in combination with LVA-pL-Gbs-rev to obtain a *sfgfp*[LVA] product. For construction of the HilE-3×Flag expression plasmid pWRG821, the *hilE* gene lacking its stop codon was PCR-amplified from STM genomic DNA with primers PtetA-HilE-Gbs-for and HilE-3×Flag-Gbs-rev. The pWSK29-based vector backbone including *tetR* and the *tetA* promoter (P*_tetA_*) was amplified from pWRG299 (Wille et al., 2012) using primers pWSK29-Gbs-for and RBS-PtetA-Gbs-rev. The fragment containing the 3×Flag tag was obtained by PCR with primers 3×Flag-Gbs-for and 3×Flag-pWSK29-Gbs-rev using pWRG447 (Wille et al., 2014) as template. The combination of all three fragments using assembly cloning resulted in pWRG821. Plasmid pWRG822 was cloned by amplifying *hilE* together with its natural promoter using primers pWSK29-PhilE-Gbs-for/HilE-3xFlag-Gbs-rev and assembly with the 3xFlag tag directly in pWSK29. For that, pWSK29 was linearized by PCR using primers pWSK29-Gbs-for/rev.

**Table 2:** Plasmids used in this study.

Plasmid	Relevant characteristic(s)	Source or reference
pMW82	pBR322 derivative, promoter-less *gfp* reporter plasmid, Ap^r^	(Bumann and Valdivia, 2007)
pWRG218	promoter-less *sfgfp*[LVA] reporter plasmid, Ap^r^	(Jennewein et al., 2015)
pWRG717	pBluescript II SK+ derivative, *aph* resistance cassette and I-SceI cleavage site, Km^r^, Ap^r^	(Hoffmann et al., 2017)
pWRG730	Heat-inducible Red recombinase expression, Tet-inducible expression of I-SceI, Cm^r^	(Hoffmann et al., 2017)
pWRG805	P*_hilC_*:*sfgfp*[LVA] in pMW82, Ap^r^	This study
pWRG806	P*_hilD_*:*sfgfp*[LVA] in pMW82, Ap^r^	This study
pWRG821	P*_tetA_*:*hilE*:3×Flag in pWSK29, Ap^r^	This study
pWRG822	P*_hilE_*:*hilE*:3×Flag in pWSK29, Ap^r^	This study
pWSK29	Low-copy-number vector, Ap^r^	(Wang and Kushner, 1991)

### Cell culture and infection

HeLa (ATCC), HuTu-80 (LGC Standards, Wesel, Germany) or C2BBe1 (ATCC) cells were grown in DMEM high (4.5 g/L) glucose (Biowest, Nuaillé, France) supplemented with 10% FCS, sodium pyruvate and 2 mM GlutaMax (Thermo Fisher Scientific, Karlsruhe, Germany) under humidified atmosphere with 5% CO_2_. Gentamicin protection assays were carried out as follows: Twenty-four hours prior infection 3 × 10^4^ HuTu-80 or 1 × 10^4^ HeLa/C2BBe1 cells per well were seeded in 96-well plates (Nunc Edge 2.0, Thermo Fisher Scientific). An inoculum corresponding to a multiplicity of infection (MOI) of 5 was prepared in pre-warmed DMEM low (1 g/L) glucose (Biowest) supplemented with 10% FCS. One hundred µL of the inoculum was added to each well and after that the plate was centrifuged for 5 minutes at 500 × *g* to synchronize infection. The plate was incubated for 10 minutes at 37°C and then the cells were washed once with DMEM. One hundred µL of DMEM containing 100 µg/mL gentamicin was applied to each well to kill remaining extracellular bacteria. After one hour of incubation the cell layers were washed twice with DMEM low glucose and then lysed for 10 min. with PBS containing 2% Elugent (Merck Millipore, Darmstadt, Germany) and 0.0625% Antifoam B (Sigma-Aldrich, Schnelldorf, Germany) to liberate the intracellular bacteria. Serial dilutions of the inoculum and the lysates were plated on Mueller Hinton (MH) plates to determine the colony-forming units. Based on the inoculum the percentage of invasive bacteria was calculated and subsequently normalized to WT.

### RNA isolation and RT-qPCR

After sampling all bacterial cultures were immediately treated either with RNA protect bacteria reagent (Qiagen, Hilden, Germany) according to manufacturer’s instructions or fixed with addition of 20% (v/v) -20°C cold STOP solution consisting of 95% (v/v) ethanol (Merck) and 5% (v/v) phenol pH4.3 (Sigma #P4682) and subsequently frozen at -80°C. RNA was isolated using the Total RNA Isolation Mini Kit (Agilent Technologies, Waldbronn, Germany) or Quick-RNA Fungal/Bacterial MiniPrep Kit (Zymo #R2014). Traces of DNA were removed by subjecting the samples to the DNA-free kit according to manufacturer’s instructions (Thermo Fisher Scientific #AM1906). Absence of DNA was tested by PCR using primers gyrB_qPCR_new-fw and –rv (Deditius et al., 2015). RT-qPCR was done using the Luna Universal One-Step RT-qPCR kit (New England Biolabs, Schwalbach, Germany) with primers targeting *hilA* (Brunelle et al., 2011), *hilC*, *hilD*, *hilE*, *iacP*, *sipA*, *sptP* and *gyrB* (**Supplementary Table S1**) on either a CFX96 (Bio-Rad, Munich, Germany) 96-well or a ViiA 7 (Thermo Fisher Scientific) 384-well real-time thermal cycler using the following cycling conditions: 55°C 15 min, 95°C 15 min, and 40 cycles of 95°C 15 sec, 60°C 15 sec, 72°C 30 sec. After normalization to the levels of the housekeeping gene *gyrB*, ΔC_t_ values, if not stated otherwise, were all normalized to normoxic controls grown in neutral LB using the ΔΔC_t_ method.

### Transcriptome analysis

RNA was isolated from pH-shifted cultures supplemented with KNO_3_ and NaOAc. For the normoxia/hypoxia comparison of STM WT transcriptome, RNA of three biological replicates was pooled and subjected to rRNA depletion, cDNA synthesis, library preparation and sequencing on a HiSeq 2500 (Illumina, Munich, Germany) in 50 bp single end mode (GATC, Konstanz, Germany). For the samples harboring pWRG821, two biological replicates were sequenced separately on a HiSeq 1500 (Illumina) in 100 bp paired end mode. Raw sequencing data can be accessed through bioproject PRJNA486717 and have been uploaded to SRA, accession number SRP158463. Reads were trimmed based on quality using Trimmomatic (Bolger et al., 2014). Rockhopper 2 (Tjaden, 2015) was used for mapping the reads to the sequence of strain *S*. Typhimurium ATCC 14028S (accessions: CP001362 + CP001363). Differential expression analysis was done within Rockhopper 2 on the basis of extended annotation data including a set of non-coding RNAs described before (Kröger et al., 2013). We calculated relative expression of hypoxia samples vs. normoxia on the basis of normalized expression values which were plotted using ggplot2 (Wickham, 2016) within R v3.4.1 (R-Core-Team, 2016). KEGG pathway and module enrichment analysis was done with the R package ‘clusterProfiler’ (Yu et al., 2012).

### Effector translocation assay

HuTu-80 cells were seeded 24 h prior infection at a density of 3 × 10^4^ in black half-area 96-well plates with transparent bottom (μClear, Greiner Bio-One, Frickenhausen, Germany). Cells were infected with bacteria grown under normoxia or hypoxia as described above but using a MOI of 200. After washing the cells twice with HBSS (Biowest) supplemented with 5% FCS, 80 µl of HBSS containing 5% FCS, 100 µg/mL gentamicin and CCF2-AM (Thermo Fisher Scientific, Karlsruhe, Germany) loading solution were applied to each well. Two hours later, ratiometric detection of the FRET substrate (excitation:409/12, emission:530/12) and the cleaved substrate (excitation:409/12, emission:460/12) was done using a M1000 plate reader (Tecan, Crailsheim, Germany) in fluorescence bottom read mode.

### Microscopy

For immunofluorescence staining, 1.8 × 10^5^ HuTu-80 cells were seeded on round coverslips within a 24-well plate. After infection samples were fixed for 15 min. using 3% PFA (Alfa Aesar) and subsequently permeabilized for 15 min. with 0.1% Triton X-100 (Sigma-Aldrich) in DPBS. Samples were blocked by 1-2 drops of Image-iT FX signal enhancer (Thermo Fisher Scientific). The F-actin cytoskeleton was stained with 1:200 diluted Oregon Green-conjugated phalloidin (Thermo Fisher Scientific) in blocking buffer (2% BSA, 2% goat serum in DPBS). After addition of 1 µg/mL DAPI (Sigma-Aldrich) in DPBS, samples were incubated protected from light for 1 h at room temperature. Samples were washed once in DPBS, ProLong Gold mounting medium (Thermo Fisher Scientific) was applied and coverslips were sealed on slides with Entellan (Merck Millipore). Slides were stored protected from light at 4°C until further use. Microscopy images were acquired with a DS-Qi1 camera attached to a Ti-E inverted microscope (both Nikon, Düsseldorf, Germany) using a 1.2 NA 63× objective and appropriate filters. Overlay of fluorescence channels was done within the NIS-Elements AR v4.1 software (Nikon).

### Protein electrophoresis and Western blot

Bacteria were grown for 3.5 h in parallel under low or ambient O_2_ as described and pellets were collected by centrifugation (8,000 × *g*, 10 min.). Sample volumes were adjusted in loading buffer (Carl Roth) to contain 0.01 OD_600_/µL. Polyacrylamide gel electrophoresis (PAGE) was carried out according to standard protocols using a Mini-Protean Tetra cell system (Bio-Rad Laboratories, Munich, Germany). Proteins were transferred to PVDF membranes (Thermo Fisher Scientific) in a Mini Trans-Blot Cell (Bio-Rad) running at 300 mA, 4°C for 2h. Antibodies against the Flag-tag (clone M2, Sigma-Aldrich, Schnelldorf, Germany) or DnaK (clone 8E2/2, Enzo Life Sciences, Lörrach, Germany) were applied in TBS with 5% skimmed milk (Carl Roth) and 3% BSA (Carl Roth) to the membranes overnight at 4°C. Bound antibodies were detected with anti-mouse horseradish peroxidase (HRP)-coupled secondary antibodies (Jackson Immuno Research/Dianova, Hamburg, Germany) using a Fusion FX Spectra chemiluminescence system (Vilber Lourmat, Eberhardzell, Germany). Band intensities were quantified using the function built in EvolutionCapt edge x64 software (Vilber Lourmat). Blot images were processed (marker overlay, tonal range, 16 to 8 bit conversion) using Photoshop Elements 15 (Adobe Systems, Munich, Germany).

## Data Availability

The datasets presented in this study can be found in online repositories. The names of the repository/repositories and accession number(s) can be found in the article/**Supplementary Material**.

## Supplementary material

Supplementary Figure 1 Expression of *hilA* and *hilD* under different growth conditions. Expression of *hilA* and *hilD* were measured by RT-qPCR using LB with media supplements as indicated, at pH 7.4 (grey) or with pH shift from 5.9 to 7.4 (green) and constant O_2_ concentrations of 11% (left) or 0.5% (right). Data shown was normalized to *gyrB* and normoxic samples grown in plain LB pH 7.4 (ΔΔC_t_, dashed line). Depicted are mean ± SD (n=4) with statistical significance calculated using a one sample *t*-Test against 0 defined as * for *p* < 0.05, ** for *p* < 0.01 and *** for *p* < 0.001.

Supplementary Figure 2 Impact of media supplements and growth conditions on *Salmonella* invasion. Invasion in HuTu-80 cells of STM WT grown under normoxia (red) or hypoxia (blue), pH 7.4 or pH shift from 5.9 to 7.4 and with media supplements as shown. A Δ*invC* mutant lacking a functional type three secretion system was included as a negative control. Statistical significance was calculated comparing normoxic and hypoxic conditions as indicated using one-way ANOVA with Holm-Šídák multiple comparisons test and was defined as ** for *p* < 0.01 and *** for *p* < 0.001. Depicted are mean ± SD (n=4-6).

Supplementary Figure 3 Expression of *hilE*. **(A)** Expression of *hilE* was measured by RT-qPCR using LB with media supplements as indicated, combined with constant O_2_ concentrations of 11% (left) or 0.5% (right). Data shown was normalized to *gyrB* and normoxic samples grown in plain LB at pH 7.4 (ΔΔC_t_, dashed line). **(B)** Expression of *hilA*, *hilD* and *hilE* were measured by RT-qPCR for STM Δ*hilE* alone, with pWSK29 [empty] or p*hilE* plasmid using normoxic (left) or hypoxic (right) growth in media supplemented with NO_3_^-^ and acetate, at pH 7.4 (grey) or with pH shift from 5.9 to 7.4 (green). Data shown was normalized to *gyrB* and depicted are mean ± SD (n=3-4) with statistical significance calculated using a one sample *t*-Test against 0 defined as * for *p* < 0.05, ** for *p* < 0.01 and *** for *p* < 0.001.

Supplementary Table 1 Oligonucleotides used in this study.

Supplementary Data Sheet 1 RNA-seq data.
