# Supplementary figures and images for: O2-dependent incapacitation of the Salmonella pathogenicity island 1 repressor HilE
Source: Front Cell Infect Microbiol. 2025 Feb 18;15:1434254. doi: 10.3389/fcimb.2025.1434254 (PMC11876186; doi:10.3389/fcimb.2025.1434254)

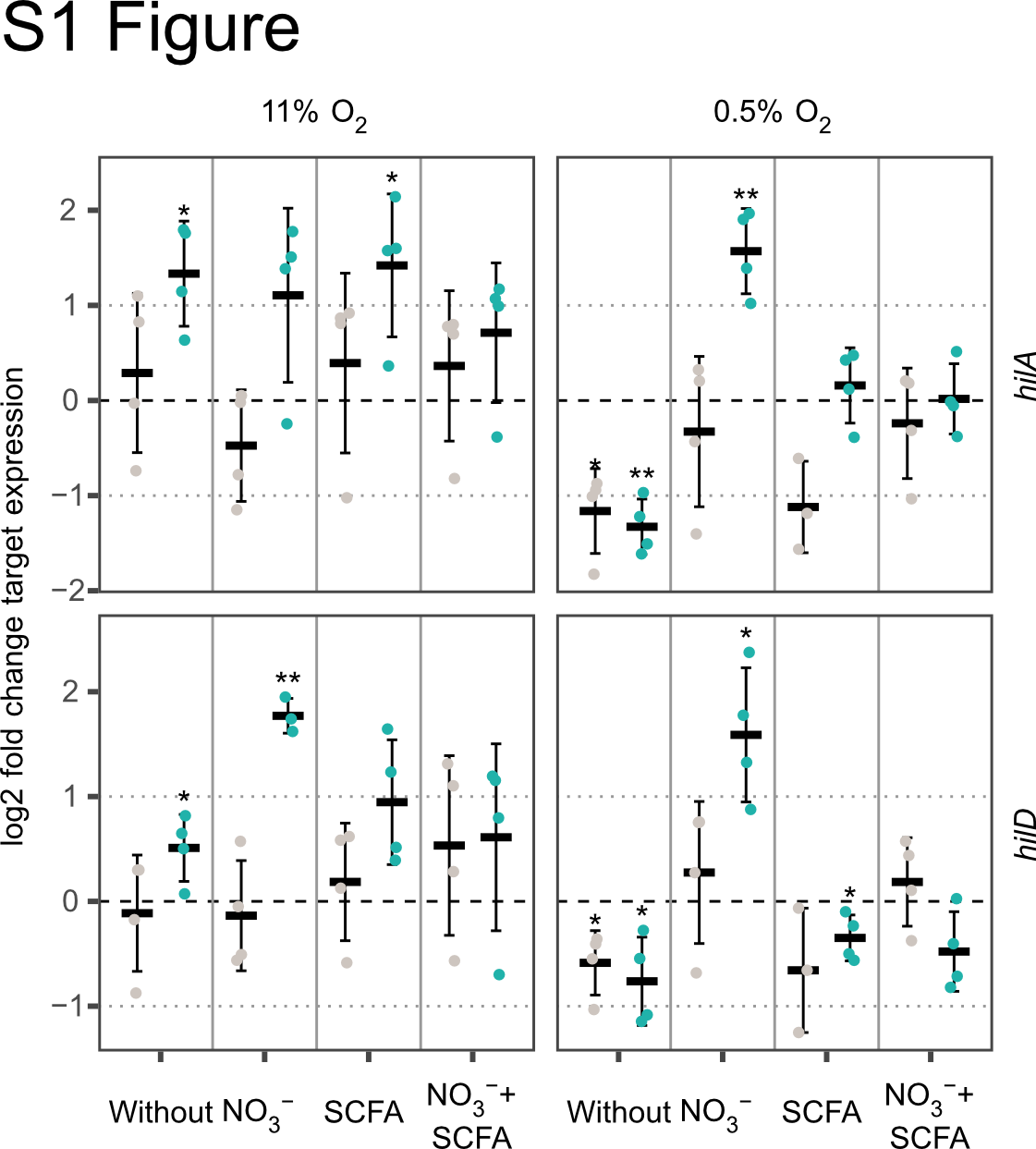

Supplement: Supplementary Figure 1 — Expression of hilA and hilD under different growth conditions. Expression of hilA and hilD were measured by RT-qPCR using LB with media supplements as indicated, at pH 7.4 (grey) or with pH shift from 5.9 to 7.4 (green) and constant O2 concentrations of 11% (left) or 0.5% (right). Data shown was normalized to gyrB and normoxic samples grown in plain LB pH 7.4 (ΔΔCt, dashed line). Depicted are mean ± SD (n=4) with statistical significance calculated using a one sample t-Test against 0 defined as * for p < 0.05, ** for p < 0.01 and *** for p < 0.001. [file Image1.tiff]

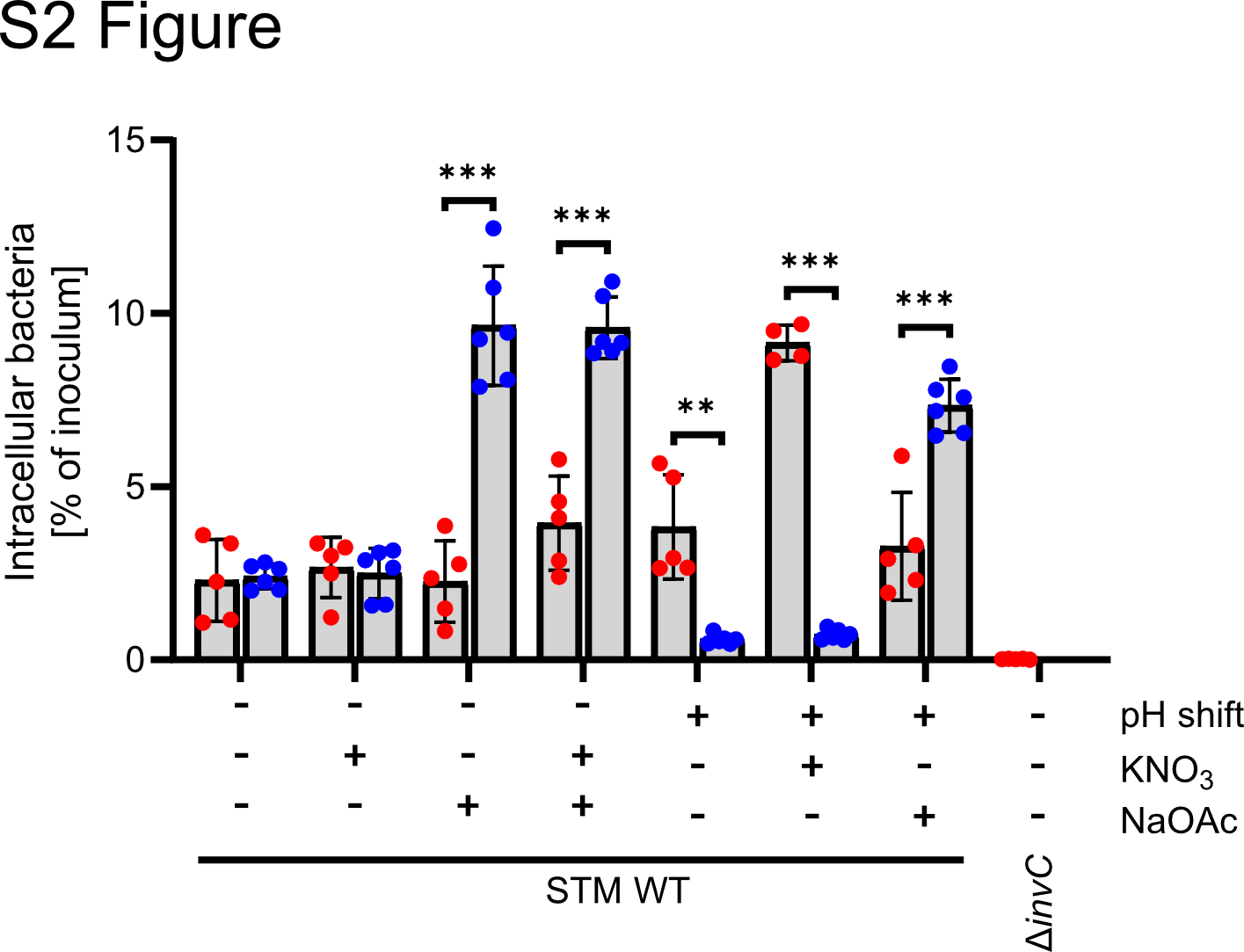

Supplement: Supplementary Figure 2 — Impact of media supplements and growth conditions on Salmonella invasion. Invasion in HuTu-80 cells of STM WT grown under normoxia (red) or hypoxia (blue), pH 7.4 or pH shift from 5.9 to 7.4 and with media supplements as shown. A ΔinvC mutant lacking a functional type three secretion system was included as a negative control. Statistical significance was calculated comparing normoxic and hypoxic conditions as indicated using one-way ANOVA with Holm-Šídák multiple comparisons test and was defined as ** for p < 0.01 and *** for p < 0.001. Depicted are mean ± SD (n=4-6). [file Image2.tiff]

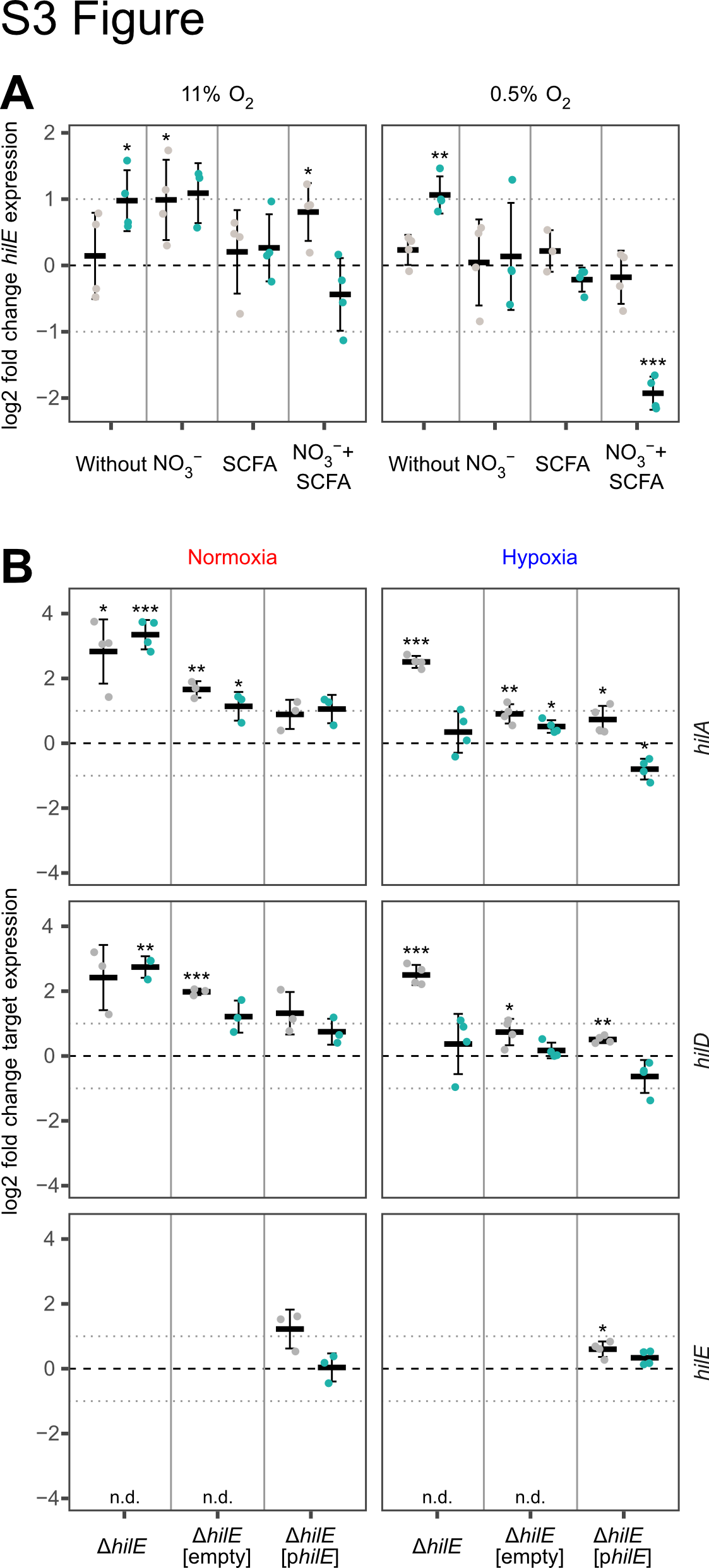

Supplement: Supplementary Figure 3 — Expression of hilE. (A) Expression of hilE was measured by RT-qPCR using LB with media supplements as indicated, combined with constant O2 concentrations of 11% (left) or 0.5% (right). Data shown was normalized to gyrB and normoxic samples grown in plain LB at pH 7.4 (ΔΔCt, dashed line). (B) Expression of hilA, hilD and hilE were measured by RT-qPCR for STM ΔhilE alone, with pWSK29 [empty] or philE plasmid using normoxic (left) or hypoxic (right) growth in media supplemented with NO3 - and acetate, at pH 7.4 (grey) or with pH shift from 5.9 to 7.4 (green). Data shown was normalized to gyrB and depicted are mean ± SD (n=3-4) with statistical significance calculated using a one sample t-Test against 0 defined as * for p < 0.05, ** for p < 0.01 and *** for p < 0.001. [file Image3.tiff]
